# A Web-Based Prediction Model for Overall Survival of Elderly Patients With Malignant Bone Tumors: A Population-Based Study

**DOI:** 10.3389/fpubh.2021.812395

**Published:** 2022-01-11

**Authors:** Jie Tang, JinKui Wang, Xiudan Pan

**Affiliations:** ^1^Department of Biostatistics and Epidemiology, School of Public Health, Shenyang Medical College, Shenyang, China; ^2^Department of Orthopedics, Children's Hospital of Chongqing Medical University, Chongqing, China

**Keywords:** nomogram, elderly patients, malignant bone tumors, overall survival, SEER

## Abstract

**Background:** Malignant bone tumors (MBT) are one of the causes of death in elderly patients. The purpose of our study is to establish a nomogram to predict the overall survival (OS) of elderly patients with MBT.

**Methods:** The clinicopathological data of all elderly patients with MBT from 2004 to 2018 were downloaded from the SEER database. They were randomly assigned to the training set (70%) and validation set (30%). Univariate and multivariate Cox regression analysis was used to identify independent risk factors for elderly patients with MBT. A nomogram was built based on these risk factors to predict the 1-, 3-, and 5-year OS of elderly patients with MBT. Then, used the consistency index (C-index), calibration curve, and the area under the receiver operating curve (AUC) to evaluate the accuracy and discrimination of the prediction model was. Decision curve analysis (DCA) was used to assess the clinical potential application value of the nomogram. Based on the scores on the nomogram, patients were divided into high- and low-risk groups. The Kaplan-Meier (K-M) curve was used to test the difference in survival between the two patients.

**Results:** A total of 1,641 patients were included, and they were randomly assigned to the training set (*N* = 1,156) and the validation set (*N* = 485). The univariate and multivariate analysis of the training set suggested that age, sex, race, primary site, histologic type, grade, stage, M stage, surgery, and tumor size were independent risk factors for elderly patients with MBT. The C-index of the training set and the validation set were 0.779 [0.759–0.799] and 0.801 [0.772–0.830], respectively. The AUC of the training and validation sets also showed similar results. The calibration curves of the training and validation sets indicated that the observed and predicted values were highly consistent. DCA suggested that the nomogram had potential clinical value compared with traditional TNM staging.

**Conclusion:** We had established a new nomogram to predict the 1-, 3-, 5-year OS of elderly patients with MBT. This predictive model can help doctors and patients develop treatment plans and follow-up strategies.

## Introduction

The most common malignant bone tumors (MBT) in elderly patients include osteosarcoma, chondrosarcoma, and chord sarcoma. Osteosarcoma is the most common malignant bone tumor in children and adolescents. About 60% of osteosarcoma patients occur under 20 years of age, and ~10% of patients occur over 60 years of age (1). Osteosarcoma usually occurs in the metaphysis of long tubular bones, most commonly around the knee joint. Osteosarcoma in adults can affect the axial and maxillofacial bones (2). Patients with osteosarcoma over 65 years of age are often secondary to Paget's disease (3). At the time of diagnosis of osteosarcoma, 10–20% of patients have already developed distant metastases, most of which are lung metastases. The prognosis of patients with osteosarcoma metastasis is inferior, and the overall 5-year survival rate is 20–30% (4). Chondrosarcoma accounts for about 9.2% of all primary MBT, with an annual incidence of 1/200,000 and an average age of onset of about 50 years old (5, 6). It is the second most common bone malignant tumor after osteosarcoma. Recent studies on chondrosarcoma report that the 5-year survival rate is about 75.2%, and the 10-year survival rate is about 70% (7, 8). Chondrosarcoma is mainly of the conventional type, and only 8–10% are of the unconventional kind. There is a big difference in survival and prognosis (9, 10). Chord sarcoma accounts for 1% to 4% of primary bone tumors, and the ratio of males to females is about 1.8:1. It can occur at any age, including children and adolescents, and is most common in patients between 50 and 60 years old (30%). Chord sarcoma grows slowly and occurs more frequently in the sacrococcygeal region (50–60%), skull base (25–35%), cervical spine (10%), thoracolumbar spine (5%), and rarely occurs in areas other than the axial bone (11). Although chord sarcoma is low to moderately malignant, 8% to 43% of patients still have distant metastasis (pulmonary metastasis is the most common), and the prognosis is poor. The overall 5-year survival rate of patients with metastatic chord sarcoma is only about 50% (11, 12).

Studies have reported that the main prognostic factors of osteosarcoma include tumor site, size, age, metastasis, metastasis site, chemotherapy, and surgery (13, 14). The significant prognostic factors of chondrosarcoma include primary tumor, histological type, tumor site, histological grade, and tumor size (15–17). One study showed that age, distant metastasis, and surgery are risk factors for chord sarcoma. However, there is no study on the prognostic factors of MBT in the elderly. There are very few elderly patients with MBT, and single-center studies cannot provide accurate estimates of prognostic factors.

Artificial intelligence has been widely used in human health care. Hassan et al. (18) summarized machine learning to predict the occurrence of sepsis to prevent patients from developing severe sepsis. Alhazmi et al. (19) used machine learning to predict the risk of oral cancer. Cho et al. (20) conducted a systematic review of the literature on machine learning to predict the risk of cancer brain metastasis.

The nomogram is a numerical model that predicts an outcome event by using estimated values generated based on various variables (21, 22). The construction of the nomogram is based on big clinical data, and a small number of cases may cause inaccurate predictions. The United States Surveillance, Epidemiology, and End Results (SEER) project is a cancer data center established in the United States in 1973, collecting information on cancer patients in 18 registries (23). The SEER database can provide enough cases to build a nomogram prediction model. Accurate prediction of survival time can help doctors better monitor patients. The prognostic factors of cancer-specific survival in elderly patients with bone tumors are not yet fully understood. Our purpose is to explore the prognostic factors affecting the survival of elderly patients with malignant bone tumors. Accurate prediction of survival time can help doctors better monitor patients. The prognostic factors of cancer-specific survival in elderly patients with bone tumors are not yet fully understood. Our purpose is to explore the prognostic factors affecting the survival of elderly patients with malignant bone tumors. We aim to construct a nomogram to predict the survival and prognosis of elderly patients with MBT to help doctors and patients formulate clinical treatment plans and follow-up strategies. After the model was established, we also conducted a series of validations on the prediction model's performance.

## Methods

### Data Source and Data Extraction

**Figure 1:**
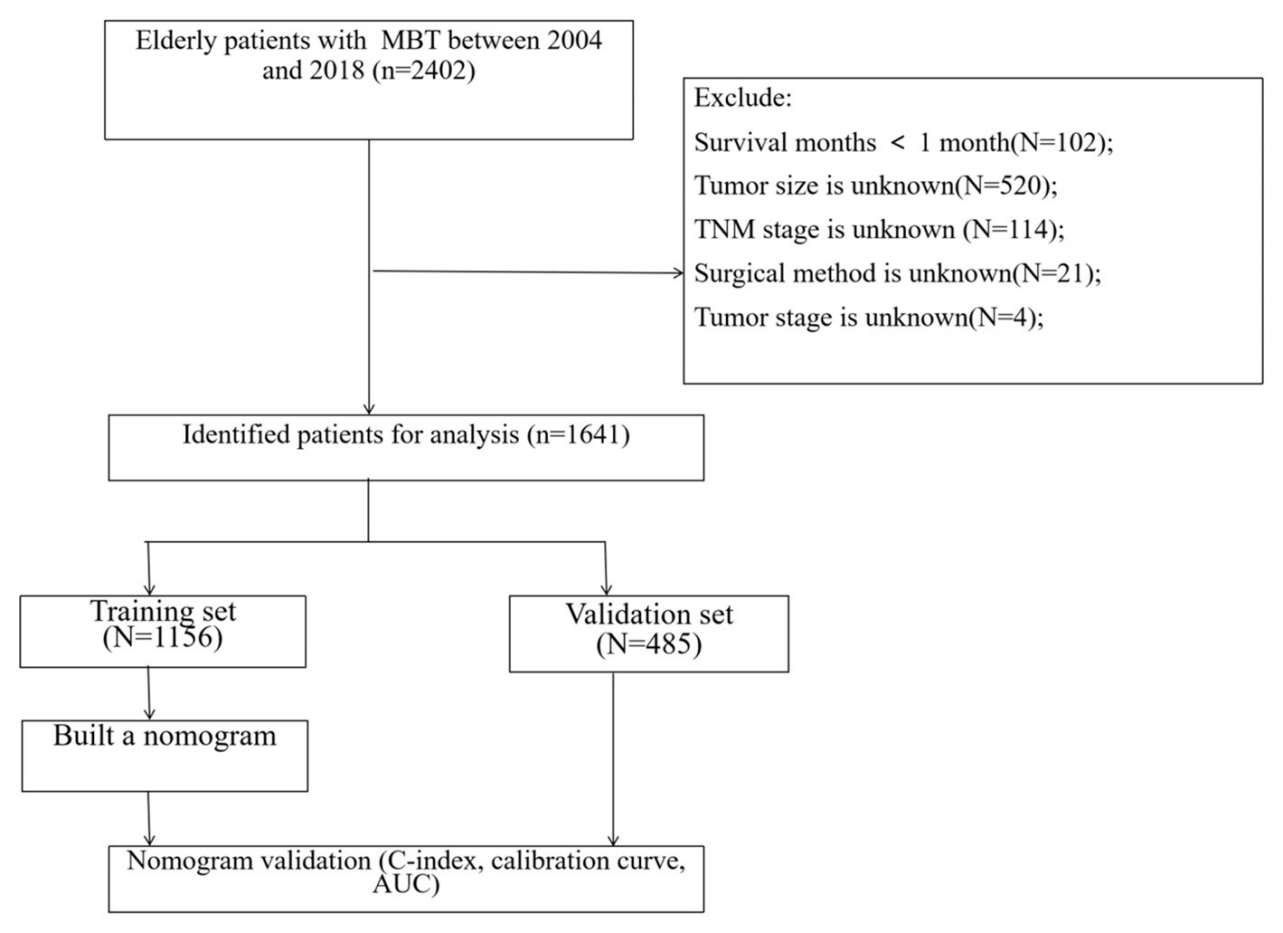
The flowchart of including and dividing patients.

The clinicopathological information of all patients over 60 years old with chondrosarcoma, osteosarcoma, and chord sarcoma from 2004 to 2018 was extracted from the SEER database. The extracted information includes age, gender, race, primary sit, histological grade, histological type, tumor size, TNM stage, surgery, radiotherapy, chemotherapy, and follow-up information. The selection criteria are: (1) age ≥ 60 years old; (2) histological classification includes: chondrosarcoma, osteosarcoma, and chord sarcoma. The exclusion criteria are: (1) survival time is less than one month; (2) tumor size is unknown; (3) TNM stage is unknown (4) surgical method is unknown; (5) tumor stage is unknown. The flow chart of patient screening is shown in Figure 1.

The age of all patients was divided into five groups, including 60–64, 65–69, 70–74, 75–79, ≥80 years old. The race was divided into three categories: white, black, and others (American Indian/AK Indian, Asian/Pacific Islander). The tumor grade was divided into four grades: well-differentiated, moderately differentiated, poorly differentiated, and undifferentiated, and some were unknown graded. The tumor stage was divided into three categories, including localized, regional, and distant. Surgical methods included no surgery, partial resection, radical resection, and amputation.

### Nomogram Construction and Validation

All patients were randomly divided into a training set (70%) and a validation set (30%). Univariate and multivariate Cox regression models were used to determine independent risk factors for patients. These risk factors were included in the nomogram to predict the 1-, 3-, and 5-year OS of elderly patients with MBT. The consistency index (C-index) was used to test the discrimination of the nomogram. The area under the receiver operating curve (AUC) was also used to evaluate the training and validation set's accuracy and discrimination. A calibration curve of 1,000 bootstrap weight samples was used to test the accuracy of the prediction model.

### Clinical Utility

Decision curve analysis (DCA) of the training set and validation set was used to evaluate the clinical value of the nomogram. A new algorithm assesses the model's weight by calculating the net benefit under the risk threshold (24). We also compared the DCA benefit of the nomogram and traditional TNM staging. According to the nomogram score, all patients were divided into low-risk and high-risk groups. Kaplan-Meier curve and the log-rank test were used to test the difference in survival between the two groups. We also compared the survival differences of patients in different risk groups under different surgical methods.

### Statistical Analysis

All statistical analyses were performed using SPSS version 26.0 and R software version 4.1.0. The median (inter-quartile range) was used to describe the data that did not follow the normal distribution for measurement data. The non-parametric test (U-test) was used to analyze the difference. For count data, used frequency (%) to describe, used chi-square analysis and non-parametric *U*-test for analysis. Univariate and multivariate Cox regression models were used to analyze survival risk factors. This study believes that P < 0.05 is statistically significant, and this test is two-sided.

## Results

### Clinical Features

A total of 1,641 patients were included, and they were randomly assigned to the training set (*N* = 1,156) and the validation set (*N* = 485). The clinical-pathological data of the patient is shown in Table 1. There were 887 males (54.1%) and 1,424 whites (86.8%). There were 958 cases (58.4%) of chondrosarcoma, 328 cases (20.0%) of osteosarcoma, and 355 cases (21.6%) of chord sarcoma. Patients with tumor grade I, II, III, and IV were 229 (14.0%), 404 (24.6%), 245 (14.9%), and 259 (15.8%), respectively; 504 (30.7%) patients were unknown grades. In the tumor stage, 713 cases (43.4%) were localized, 696 cases (42.4%) were regional, and 232 cases (14.1%) were distant. There were 336 (20.5%) without surgery, 458 (27.9%) with partial resection, 661 (40.3%) with radical resection, and 186 (11.3%) with amputation. Tumor primary sites included 623 cases (38.0%) in limbs, 231 cases (14.1%) in skull, 134 (8.17%) in spine, 246 cases (15.0%) in thorax, and 407 cases (24.8%) in pelvis. There is no significant difference between the clinical-pathological data of the patients in the training set and the validation set.

**Table 1:** Clinicopathological characteristics of elderly patients with MBT.

	**Total**	**Training set**	**Validation set**	***P***
	***N* = 1,641**	***N* = 1,156**	***N* = 485**	
Age				0.054
60–64	416 (25.4%)	276 (23.9%)	140 (28.9%)	
65–69	376 (22.9%)	285 (24.7%)	91 (18.8%)	
70–74	338 (20.6%)	233 (20.2%)	105 (21.6%)	
75–79	223 (13.6%)	155 (13.4%)	68 (14.0%)	
≥80	288 (17.6%)	207 (17.9%)	81 (16.7%)	
Race				0.141
White	1,424 (86.8%)	1,015 (87.8%)	409 (84.3%)	
Black	94 (5.73%)	63 (5.45%)	31 (6.39%)	
Other	123 (7.50%)	78 (6.75%)	45 (9.28%)	
Sex				0.716
Male	887 (54.1%)	621 (53.7%)	266 (54.8%)	
Female	754 (45.9%)	535 (46.3%)	219 (45.2%)	
Year of diagnosis				0.710
2004–2010	613 (37.4%)	428 (37.0%)	185 (38.1%)	
2010–2018	1,028 (62.6%)	728 (63.0%)	300 (61.9%)	
Histologic type				0.258
Chondrogenic sarcoma	958 (58.4%)	687 (59.4%)	271 (55.9%)	
Osteogenic sarcoma	328 (20.0%)	231 (20.0%)	97 (20.0%)	
Chordal sarcoma	355 (21.6%)	238 (20.6%)	117 (24.1%)	
Grade				0.278
I	229 (14.0%)	153 (13.2%)	76 (15.7%)	
II	404 (24.6%)	295 (25.5%)	109 (22.5%)	
III	245 (14.9%)	181 (15.7%)	64 (13.2%)	
IV	259 (15.8%)	182 (15.7%)	77 (15.9%)	
Unknown	504 (30.7%)	345 (29.8%)	159 (32.8%)	
Stage				0.174
Localized	713 (43.4%)	503 (43.5%)	210 (43.3%)	
Regional	696 (42.4%)	501 (43.3%)	195 (40.2%)	
Distant	232 (14.1%)	152 (13.1%)	80 (16.5%)	
Primary site				0.123
Limb	623 (38.0%)	444 (38.4%)	179 (36.9%)	
Cranial	231 (14.1%)	161 (13.9%)	70 (14.4%)	
Spine	134 (8.17%)	103 (8.91%)	31 (6.39%)	
Thoracic	246 (15.0%)	179 (15.5%)	67 (13.8%)	
Pelvic	407 (24.8%)	269 (23.3%)	138 (28.5%)	
T				0.141
T1	940 (57.3%)	680 (58.8%)	260 (53.6%)	
T2	652 (39.7%)	446 (38.6%)	206 (42.5%)	
T3	38 (2.32%)	24 (2.08%)	14 (2.89%)	
T4	11 (0.67%)	6 (0.52%)	5 (1.03%)	
N				1.000
N0	1,605 (97.8%)	1,131 (97.8%)	474 (97.7%)	
N1	36 (2.19%)	25 (2.16%)	11 (2.27%)	
M				0.899
M0	1,486 (90.6%)	1,048 (90.7%)	438 (90.3%)	
M1	155 (9.45%)	108 (9.34%)	47 (9.69%)	
Tumor size (median [IQR])	70.0 [44.0, 112.0]	70.0 [43.0, 112.0]	71.0 [45.0, 111.0]	0.3598
Chemotherapy				0.975
No/Unknown	1,395 (85.0%)	982 (84.9%)	413 (85.2%)	
Yes	246 (15.0%)	174 (15.1%)	72 (14.8%)	
Radiation				0.986
No/Unknown	1,227 (74.8%)	865 (74.8%)	362 (74.6%)	
Yes	414 (25.2%)	291 (25.2%)	123 (25.4%)	
Surgery				0.767
No	336 (20.5%)	236 (20.4%)	100 (20.6%)	
Partial resection	458 (27.9%)	325 (28.1%)	133 (27.4%)	
Radical resection	661 (40.3%)	470 (40.7%)	191 (39.4%)	
Amputation	186 (11.3%)	125 (10.8%)	61 (12.6%)	

### Univariate and Multivariate Cox Regression Analysis

We used univariate Cox regression analysis to confirm the risk factors for the prognosis initially. The selected risk factors were then included in the multivariate Cox regression analysis. In the end, we found that age, sex, race, primary site, histologic type, grade, stage, M stage, surgery, and tumor size were independent risk factors for elderly patients with MBT. The independent risk factors screened by univariate and multivariate analysis are shown in Table 2.

**Table 2:** Univariate and multivariate analyses of OS in the training set.

	**Univariate**			**Multivariate**		
	**HR**	**95% CI**	***P***	**HR**	**95% CI**	***P***
Age						
60–64	Reference			Reference		
65–69	1.06	0.74–1.52	0.76	1.19	0.91–1.56	0.20
70–74	0.87	0.59–1.28	0.48	1.13	0.85–1.50	0.41
75–79	1.27	0.84–1.94	0.26	2.06	1.53–2.76	<0.001
≥80	1.51	1.03–2.22	0.03	2.29	1.764–3.00	<0.001
Race						
White	Reference			Reference		
Black	1.25	0.74–2.13	0.4	0.98	0.69–1.41	0.93
Other	0.61	0.35–1.06	0.08	0.60	0.41–0.88	0.01
Sex						
Male	Reference			Reference		
Female	0.76	0.59–0.97	0.03	0.84	0.70–0.99	0.04
Year of diagnosis						
2004–2010	Reference					
2010–2018	0.56	0.43–0.72	<0.001			
Histologic type						
Chondrogenic sarcoma	Reference			Reference		
Osteogenic sarcoma	2.78	2.04–3.78	<0.001	1.25	0.98–1.60	0.08
Chordal sarcoma	0.7	0.49–0.99	0.04	0.61	0.434–0.86	0.01
Primary site						
Limb	Reference			Reference		
Cranial	0.56	0.37–0.84	<0.001	1.19	0.86–1.63	0.30
Spine	0.86	0.54–1.35	0.51	2.05	1.45–2.90	<0.001
Thoracic	0.32	0.21–0.5	<0.001	0.57	0.41–0.77	<0.001
Pelvic	0.97	0.7–1.32	0.83	0.98	0.77–1.26	0.89
Grade						
I	Reference			Reference		
II	1.77	1.02–3.06	0.04	1.19	0.84–1.69	0.34
III	5.39	3.1–9.37	<0.001	2.5	1.76–3.65	<0.001
IV	10.64	6.1–18.57	<0.001	2.88	1.969–4.22	<0.001
Unknown	2.14	1.26–3.64	<0.001	1.71	1.161–2.52	0.01
Stage						
Localized	Reference			Reference		
Regional	2.22	1.66–2.98	<0.001	1.61	1.31–1.98	<0.001
Distant	8	5.36–11.95	<0.001	1.79	1.17–2.72	0.007
T						
T1	Reference					
T2	2.95	2.27–3.83	<0.001			
T3	2.57	1.12–5.9	0.03			
T4	0	0–9.8	0.97			
N						
N0	Reference					
N1	1.77	0.79–3.93	0.16			
M						
M0	Reference			Reference		
M1	7.47	4.78–11.68	<0.001	1.623	1.04–2.53	0.03
Surgery						
No	Reference			Reference		
Partial resection	0.33	0.23–0.48	<0.001	0.429	0.33–0.56	<0.001
Radical resection	0.43	0.31–0.59	<0.001	0.397	0.31–0.51	<0.001
Amputation	0.71	0.46–1.11	0.14	0.36	0.26–0.50	<0.001
Radiation						
No/Unknown	Reference					
Yes	1.41	1.07–1.87	0.02			
Chemotherapy						
No/Unknown	Reference					
Yes	3.56	2.56–4.96	<0.001			
Tumor size	1.01	1.01–1.01	<0.001	1.00	1.00–1.01	<0.001

### Nomogram Construction and Validation

**Figure 2:**
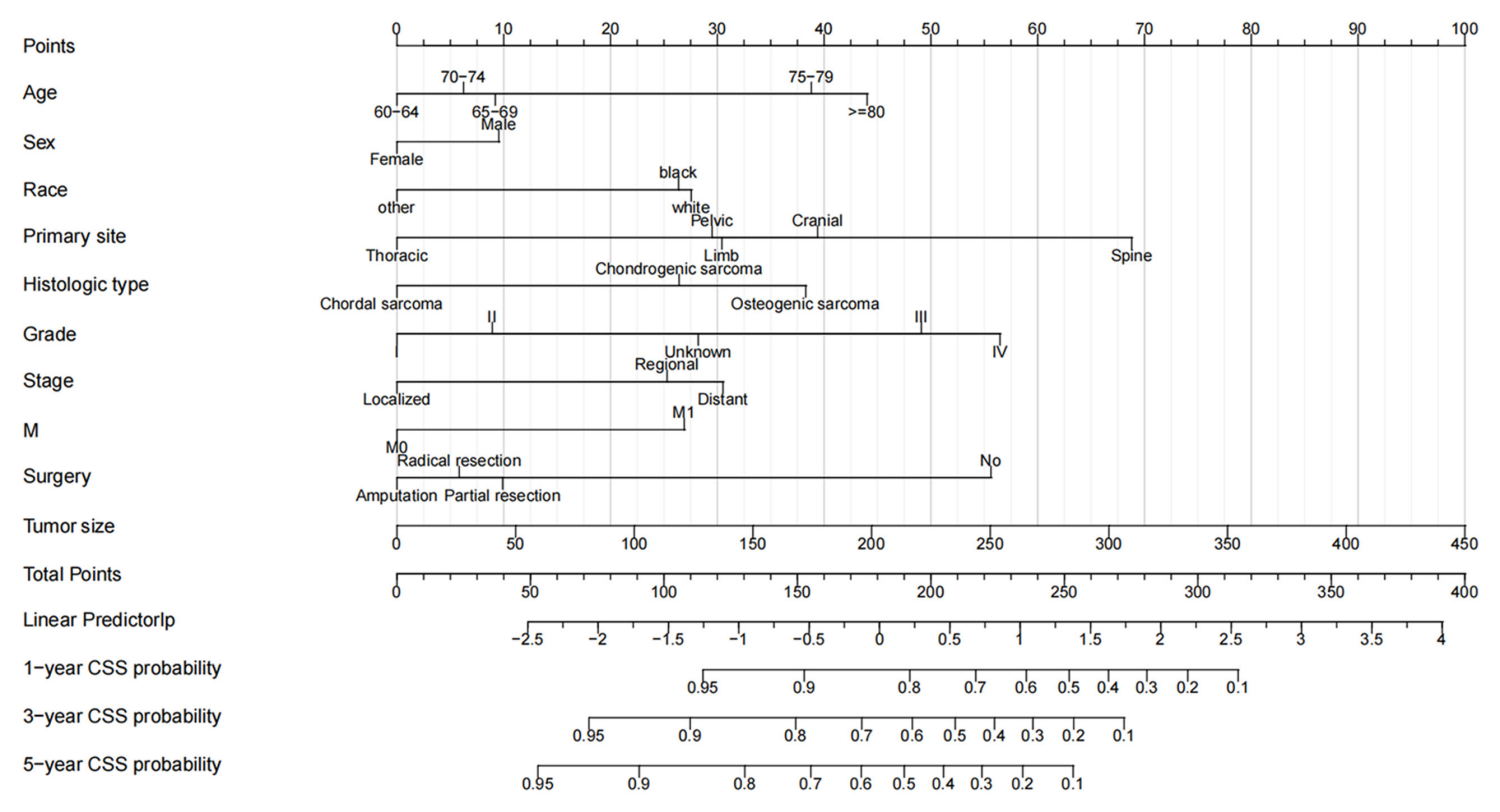
Nomogram for 1-, 3-, and 5-year OS of elderly patients with MBT. The first row of the nomogram is the scoring ruler, rows 2–11 are the variables, and the 12th row is the patient's total score. 13–16 are the full score corresponding to the patient's 1-, 3-, and 5- survival rates.

**Figure 3:**
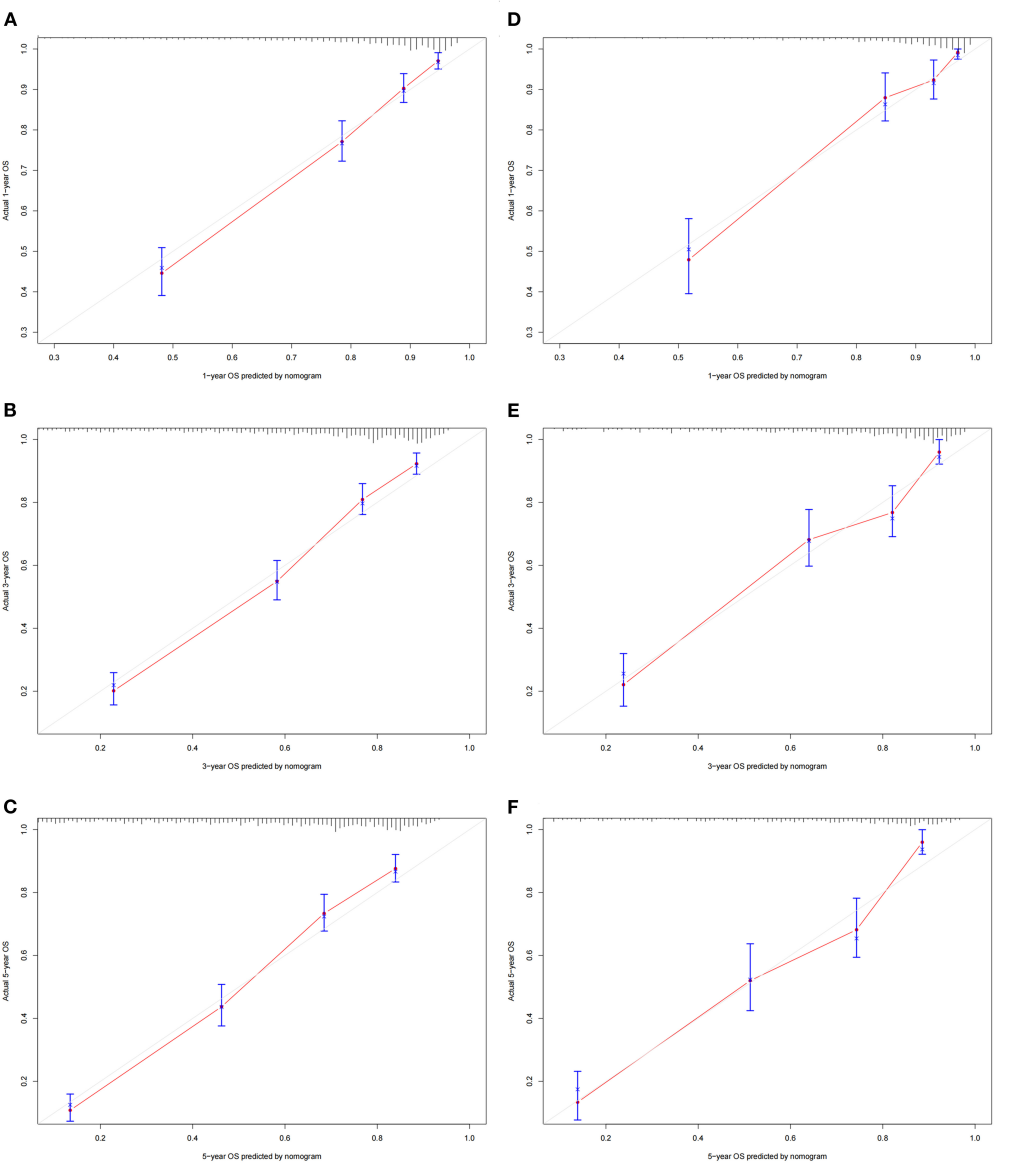
Calibration curves of the nomogram. **(A–C)** For 1-, 3-, and 5-year OS in the training set; **(D–F)** For 1-, 3-, and 5-year OS in the validation set. The horizontal axis is the predicted value of the nomogram, and the vertical axis is the actual observed value of the nomogram. The coincidence between the predicted curve and the diagonal line means that the predicted value and the actual observed value are almost the same.

**Figure 4:**
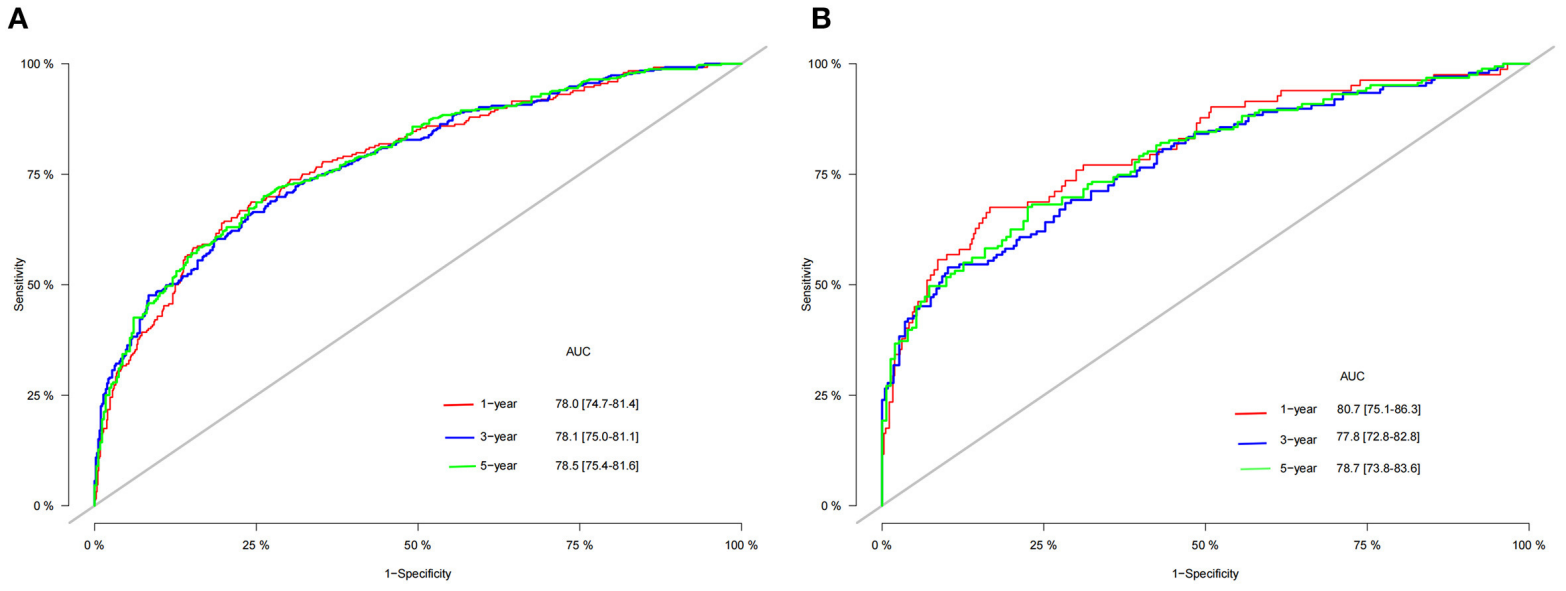
The AUC of 1-, 3- and 5-year training set **(A)** and validation set **(B)**.

A nomogram was constructed based on the identified independent risk factors to predict the 1-, 3-, and 5-year OS of elderly patients with MBT (Figure 2). It can be seen from the nomogram that tumor size has the most significant impact on OS, followed by primary tumor site, grade, and surgery. The calibration curves of the training set and the validation set showed that the observed value and the predicted value were highly consistent, indicating the accuracy of the nomogram prediction (Figure 3). The C-index of the training set and the validation set were 0.779 [0.759–0.799] and 0.801 [0.772–0.830], respectively, indicating that the nomogram had good discrimination. The AUC of the training set and the validation set also showed similar results (Figure 4).

### Clinical Application of the Nomogram

**Figure 5:**
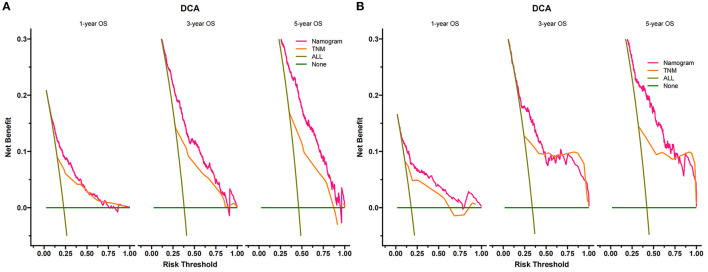
Decision curves of the nomogram predicting OS in the training set **(A)** and validation set **(B)**. The y-axis represents the net benefit, and the x-axis represents the threshold probability. The green line indicates that no patients have died, and the dark green line indicates that all patients have died. When the threshold probability is between 20 and 100%, the net benefit of the model exceeds all deaths or no deaths.

**Figure 6:**
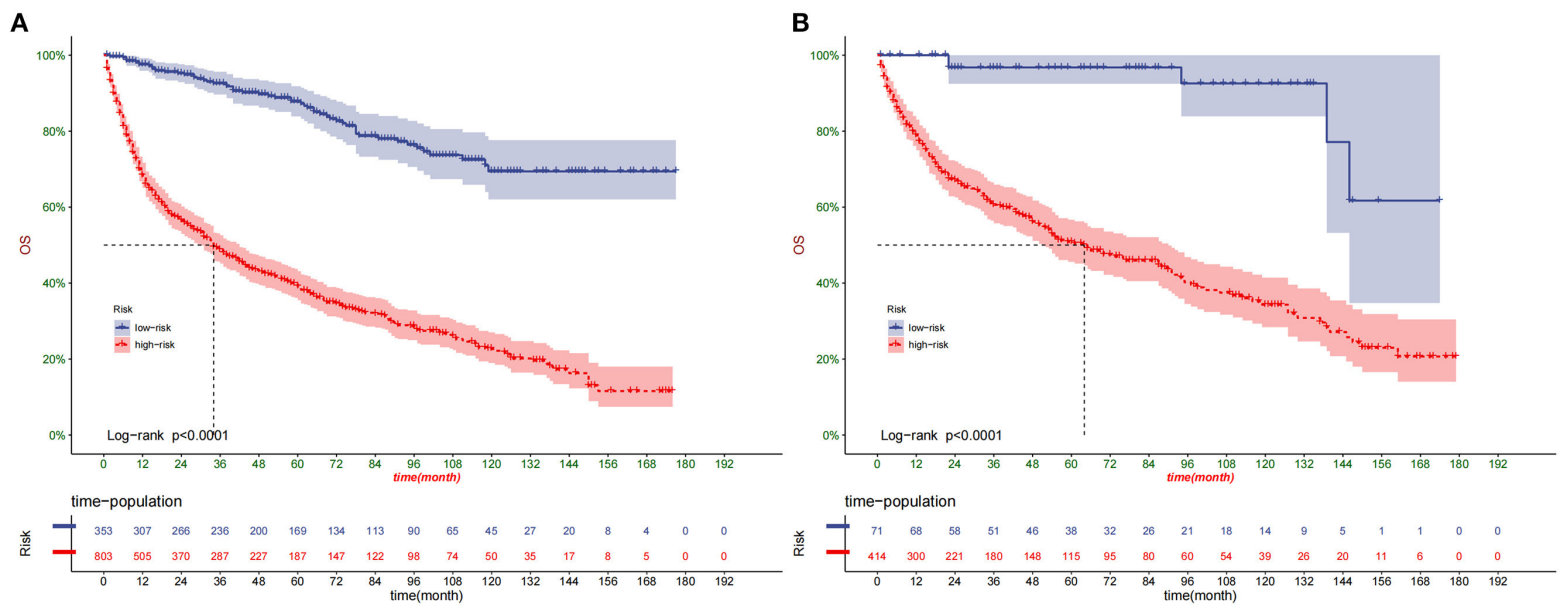
Kaplan–Meier curves of OS for patients in the low- and high-risk groups in the training set **(A)** and validation set **(B)**.

**Figure 7:**
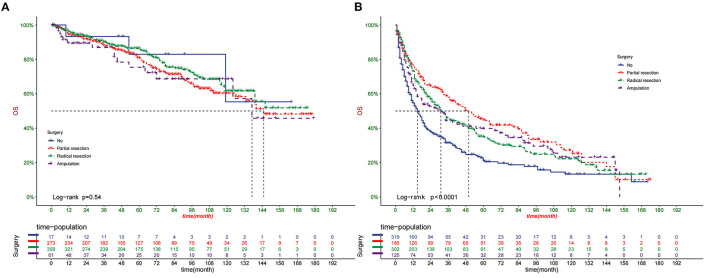
Comparison of different surgical methods of Kaplan–Meier curves in low-risk **(A)** and high-risk **(B)** groups.

DCA suggested that nomogram had potential clinical value compared with traditional TNM staging (Figure 5). Based on the score of each patient in the nomogram, the patients were divided into a low-risk group (total score ≤ 149.4) and a high-risk group (total score > 149.4). Apparent differences in survival were observed from the K-M curves of the training set and validation sets' K-M curves (Figure 6). The K-M curve indicated that the nomogram has an excellent discriminating ability. We found that patients in the high-risk group who chose partial resection had a higher survival rate according to risk groups. At the same time, there is no significant difference in the prognosis of patients in the low-risk group with various treatment methods (Figure 7).

### Online Application for OS Prediction

We have developed a web calculator based on the nomogram, which can be accessed at https://jietang.shinyapps.io/DynNomapp/. Enter the clinicopathological characteristics of the patient on this web page to get the corresponding survival probability. This calculation is easy to use and friendly to both patients and doctors.

## Discussion

Cancer is one of the most common causes of death in elderly patients. The morbidity and mortality of bone tumors are high. A study has reported 3,600 new bone tumor patients in the United States each year and 1,720 deaths from bone tumors (25). Although MBT is not common, their mortality is still high. The three most common MBT in elderly patients include chondrosarcoma, osteosarcoma, and chord sarcoma, of which chondrosarcoma is the most common (5, 6). Our study also found that most elderly patients with MBT over 60 years old are chondrosarcoma. Giuffrida et al. (7) found considerable differences in the 5-year survival rate of patients with different chondrosarcoma subtypes. The 5-year survival rate of dedifferentiated chondrosarcoma is 0%, while the exact cell type reaches 100%, the mucinous type is 71%, the paracortical type is 93%, the mesenchymal chondrosarcoma is 48%, and the malignant chondroblastoma is 85%. Strotman et al. (10) found that the 5-year overall survival rate of dedifferentiated chondrosarcoma is only 18%. The prognosis is worse for patients with axial bone, tumor size >8 cm, and lung cancer metastasis. A study by Bielack et al. (2) showed that age, location, and metastasis are the prognostic factors of patients with osteosarcoma. Previous studies have reported the predictive factors of osteosarcoma and predicted overall survival and cancer-specific survival (26, 27). Chord sarcoma is also a malignant bone tumor with high incidence in elderly patients. Previous studies have reported that chordoma mainly occurs in patients over 30 years old, and most of them are people over 60 years old (28). Chord sarcoma rarely metastasizes, but the 5-year survival rate will drop to about 50% (12). The incidence and prognostic factors of MBT in elderly patients have not yet been reported in the literature. Accurate prediction of patient survival is conducive to future treatment and follow-up. Therefore, we developed and validated nomograms to predict the survival of elderly patients with MBT.

In our study, we found that the increase in age will reduce the survival rate of elderly patients. Previous studies have also reported that age is a risk factor for bone tumors (27, 29). The reason may be that the increase in age leads to a decrease in the suppression of tumors by the immune system and an increase in the probability of comorbidities. In addition, our study also found that the tumor site and size are also important factors affecting the prognosis. Similar to previous studies, patients with axial and large tumors have a higher risk of death (30–32). It may be because larger tumors are more likely to metastasize, and tumors in the axial position, especially the spine, are more likely to metastasize and invade surrounding tissues (33). Previous studies have found that the tumor stage is also a significant risk factor. The prognosis of distantly metastatic tumors is worse than localized tumors (34, 35). Our study also found that the tumor stage is an independent risk factor for prognosis. Besides, we found that sex and race are also significant risk factors, which have not been seen in previous studies. Women have a better survival prognosis than men. American Indians and Asians seem to have higher survival rates than whites and blacks.

In this study, we found that surgery significantly improved the prognosis of patients, similar to the results of previous studies (36, 37). In addition, according to risk stratification, we found that patients in the high-risk group benefited the most from partial tumor resection. At the same time, there is no significant difference in the prognosis of patients in the low-risk group with various treatment methods. Since most of the patients in the high-risk group are in the late stage of the disease and have already developed distant metastases, radical resection does not seem to improve the prognosis of the patients. This has some enlightenment for doctors and patients in choosing surgical methods for patients with different risks. On chemotherapy, our study found that chemotherapy does not affect patient survival, similar to previous studies (38).

Although our nomogram includes three histological types of MBT, it has been validated that the prediction model has good accuracy and discrimination. The C-index of the training set and the validation set were 0.779 [0.759–0.799] and 0.801 [0.772–0.830], respectively, proving the discriminative ability of the nomogram. The predicted value of the calibration curve is highly consistent with the observed value, which indicates the accuracy of the prediction model. We compared the 1-, 3-, and 5-year DCA of the nomogram with the traditional TNM staging system and proved that the clinical value of the nomogram is higher than that of the traditional TNM staging system. Risk stratification also accurately distinguished high-risk and low-risk patients, and treatment and follow-up strategies should be different for patients in other risk groups. Patients in the high-risk group should choose appropriate treatment and receive closer follow-up.

This study is the first to explore risk factors for death in elderly patients with MBT using data from the SEER database. The overall survival rate of elderly patients with MBT was predicted based on risk factors. In other words, when elderly patients with MBT are admitted to hospitals, doctors can accurately inform patients of the 1-, 3- and 5-year survival rates based on these critical prognostic factors and make corresponding treatment decisions and follow-up strategies according to the survival rate of patients. This nomogram is of great significance for elderly patients with MBT.

However, our study still has some limitations. First, the SEER database cannot obtain detailed information on some variables such as surgical margins, BMI, smoking, and drinking, so it has a specific impact on prediction accuracy. However, we include essential variables such as tumor stage, size, surgery, and other factors that affect the prognosis of patients so that the results will not cause a devastating deviation. Second, The lack of information on some critical variables will cause errors in the prediction results. For example, the tumor grade of some patients is unknown. However, it can be seen from the nomogram that the lack of tumor grade of some patients did not cause serious bias. Finally, our nomogram has only been validated internally, and external validation is necessary to test the predictive power of the nomogram.

## Conclusion

A comprehensive nomogram was constructed to predict the 1-, 3-, and 5-year overall survival of elderly patients with MBT. This nomogram has been validated to have good accuracy and reliability and help patients and doctors predict survival prognosis and formulate treatment and follow-up strategies.

## Data Availability Statement

Publicly available datasets were analyzed in this study. This data can be found here: https://seer.Cancer.gov.

## Ethics Statement

The public and anonymous data shared in this study is obtained from the SEER database.

## Author Contributions

JT, JW, and XP: contributed to the conception and design. JW and JT: collected, analyzed the data, drew the figures and tables, and wrote the draft. XP and JT contributed to manuscript writing and revision. All authors approved the final manuscript.

## Conflict of Interest

The authors declare that the research was conducted in the absence of any commercial or financial relationships that could be construed as a potential conflict of interest.

## Publisher's Note

All claims expressed in this article are solely those of the authors and do not necessarily represent those of their affiliated organizations, or those of the publisher, the editors and the reviewers. Any product that may be evaluated in this article, or claim that may be made by its manufacturer, is not guaranteed or endorsed by the publisher.
